# Polycystic Ovary Syndrome May Be an Autoimmune Disorder

**DOI:** 10.1155/2016/4071735

**Published:** 2016-05-05

**Authors:** Hifsa Mobeen, Nadeem Afzal, Muhammad Kashif

**Affiliations:** Department of Immunology, University of Health Sciences, Lahore 54600, Pakistan

## Abstract

Polycystic ovarian syndrome (PCOS) is the most prevalent endocrine disorder affecting females. It is a common cause of menstrual irregularities and infertility during reproductive age. Genetic and hormonal factors play crucial role in the pathogenesis of PCOS. Low level of progesterone in PCOS causes overstimulation of immune system that produces more estrogen which leads to various autoantibodies. Different autoantibodies have been documented in PCOS, for example, anti-nuclear (ANA), anti-thyroid, anti-spermatic, anti-SM, anti-histone, anti-carbonic anhydrase, anti-ovarian, and anti-islet cell antibodies. There is an association between PCOS and autoimmune diseases such as ANA and anti-TPO that have been documented in systemic lupus erythematosus and Hashimoto thyroiditis, respectively, and it is suspected that there are autoantibodies that might affect the long term clinical management of these patients. Therefore fluctuating levels of autoantibodies in different PCOS patients give us the way to open new chapter for future research on molecular level. This may lead to discovery of better treatment options for PCOS in near future.

## 1. Polycystic Ovarian Syndrome

In women polycystic ovarian syndrome (PCOS) was first described in 1935, by Stein and Leventhal [1, 2]. PCOS is the most common cause of menstrual disturbance such as oligomenorrhea, anovulation, menorrhagia, and infertility [3].

PCOS was estimated to be 4–8% in Greece, Spain, and the USA. Throughout the world its prevalence is increasing and is showing galloping increase in parallel with type 2 diabetes mellitus (T2DM) [4]. Worldwide there were 116 million women affected by PCOS [5]. In Pakistan about 5%–10% of women were affected by PCOS in 2009 [6]. Different signs and symptoms of PCOS along with their frequencies are shown in Table 1.

**Figure 1:**
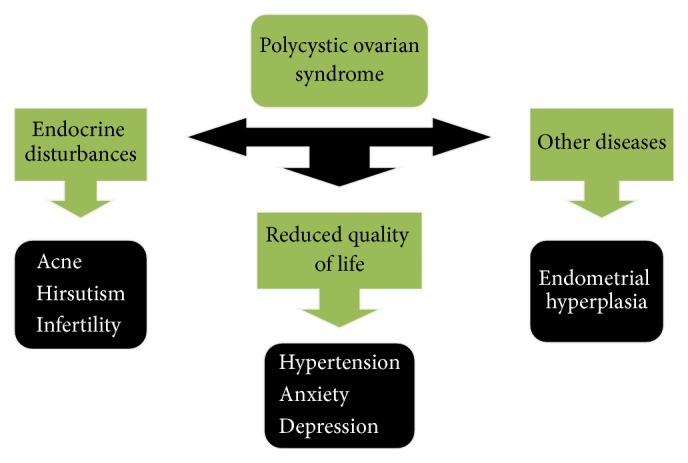
Flow chart showing possible consequences and outcomes of PCOS.

Regarding pathophysiology, PCOS is a heterogeneous disorder characterized by ovulatory dysfunction, hyperandrogenism, and polycystic ovarian morphology. Its characteristic neuroendocrine features include increased serum concentration of luteinizing hormone (LH), increased LH/FSH ratio, and increase in amplitude and frequency of pulsatile LH secretion (Figure 1) [7–9].

Hypothalamus secretes gonadotrophin releasing hormone (GnRH) which binds its receptors on secretory cells of adenohypophysis [10, 11]. In response to GnRH, gonadotrophs produce LH and FSH, which regulate development, growth, pubertal maturation, and reproductive processes of body [12].

In females FSH and LH activate ovaries to produce estrogen and inhibin, to regulate menstrual cycle. Estrogen forms a negative feedback loop by inhibiting production of GnRH by hypothalamus [12].

PCOS can affect not only females but also males but with less frequency. Although men do not have ovaries but underlying defects (high levels of androgens and low level steroid binding globulin) and clinical features of PCOS can also be seen in males and they are referred to as Stein-Leventhal syndrome (Figure 1) [13–15].

The level of progesterone is decreased in PCOS which cannot suppress GnRH/LH pulse frequency in PCOS; therefore, increased estrogen secretion may lead to autoantibodies, for example, anti-nuclear, anti-thyroid, and anti-islet cell antibodies [16].

Currently there are two widely accepted diagnostic criteria of PCOS and both suggest presence of two out of three signs to be labeled as PCOS (Table 2) [17–19].

Obesity exacerbates comorbidities of PCOS such as hypertension, diabetes, hypercholesterolemia, and heart disease [20, 21]. An ovulation in PCOS leads to unopposed estrogen secretion which is a risk factor for endometrial hyperplasia and carcinoma. PCOS reduces quality of life by depression, anxiety, obesity, infertility, and hirsutism [22]. Kerchner et al. detected depression in 40% of women of PCOS and the incidence of suicide is increased up to 7-fold in PCOS (Figure 1) [23, 24].

## 2. Autoimmunity: Could It Be a Causative Factor for PCOS?

In autoimmunity there is breakdown of mechanisms responsible for self-tolerance and there is induction of an immune response against self-components. Autoimmunity is characterized by induction of autoreactive cells (e.g., B cells, T cells) and proteins (e.g., antibodies). Autoimmunity is classified as organ specific and nonorgan specific autoimmunity [25].

Examples of organ specific autoimmunity include Grave's disease, Hashimoto's thyroiditis, and IDDM [26] whereas examples of systemic autoimmunity are SLE, rheumatoid arthritis, rheumatic fever, and so forth [27].

## 3. Etiology of Autoimmune Diseases 

Although exact reason for autoimmunity is not known, various mechanisms have been suggested for its development as follows.

### 3.1. Sequestered Antigens

Lymphoid cells may not be exposed to some of the self-antigens during their differentiation. The release of antigen from these organs due to accidental trauma, injury, or surgery can result in the stimulation of an immune response and initiation of autoimmune diseases, for example, sperms and neuron cells [39].

### 3.2. Molecular Mimicry

When environmental substances that resemble our body components are exposed to the body, the immune system generates response against these substances which cross-react with body's own tissue; for example, coxsackievirus has molecular mimicry with *β* cells of pancreas [40].

### 3.3. Alteration of Normal Protein

Drugs can bind to normal proteins and make them immunogenic; for example, methyldopa binds to RBC's surface proteins and causes autoimmune haemolytic anaemia [41].

### 3.4. Failure or Decrease of T Regulatory Cells (Tregs)

Tregs are characterized by the expression of CD4, CD25, and FOXP3. They suppress proinflammatory effects of other T cells by producing IL-10 and play role in peripheral tolerance of autoreactive T cells. If there is failure or decrease of Tregs, then autoreactive cells will not be killed and ultimately may lead to autoimmunity [42].

In PCOS there is an excess of estrogen which has been linked to different autoimmune diseases. Estrogen increases production of IL-4, IL-1, IL-6, and interferon-*γ*.

## 4. Autoantibodies in PCOS

### 4.1. Anti-Nuclear Antibody (ANA)

Inflammation, immune hyperstimulation, and process of tissue destruction expose intracellular antigens that lead to production of ANA which is a hallmark of autoimmune disorders [43]. ANA has been detected in many autoimmune disorders such as systemic lupus erythematosus, Sjogren's syndrome, polymyositis, dermatomyositis, and autoimmune hepatitis [43–46].

### 4.2. Anti-Thyroid Antibody

Autoantibodies against one or more components of thyroid are produced in autoimmune thyroiditis. Anti-thyroid antibodies include anti-thyroid peroxidase (anti-TPO), thyrotrophic receptor (TRAbs), and thyroglobulin antibodies [47]. Anti-TPO antibodies are associated with Hashimoto thyroiditis [48–51]. CD4 T cells produce INF-*γ* which induces MHC-II on thyroid cells that expands autoreactive T cells and prolongs inflammatory response [51].

Patrikova et al. has suggested strong association of anti-thyroid antibodies with PCOS, for example, anti-TPO 7.81% [52]. Kachuei et al. reported strong association of anti-thyroglobulin (*p* = 0.275) and anti-TPO antibodies (*p* = 0.040) in PCOS patients [53]. Arduc et al. suggested association of anti-thyroglobulin (*p* = 0.039) and anti-TPO antibodies (*p* = 0.002) in PCOS [54]. Janssen et al. suggested that autoimmune thyroiditis (AIT) is three times more common in PCOS as compared to non-PCOS women of reproductive age [55]. Sarkar reported strong association of infertility, miscarriages, and disturbed thyroid profile in pregnant females. Both hypo- and hyperthyroidism can lead to increased rate of miscarriages, fetal death, and late cognitive development of offsprings [56].

### 4.3. Anti-Islet Cell Antibody

Islet cell autoantibodies are produced when beta cells of pancreas are damaged and they can bind to glutamic acid decarboxylase (GAD), protein tyrosine phosphatase, islet antigen-2 (IA-2), insulin, and zinc transporter (ZNT8) and lead to further destruction of islet cells of pancreas [63]. The destruction of beta cells of pancreas causes hyperglycemia, which can be treated by insulin therapy to control hyperglycemia, but it leads to increase in weight gain as well as ovarian hyperandrogenism [64].

Islet cell autoantibodies react with islet cell antigens in particular sequence reacting with insulin or GAD first, followed by IA-2 and ZNT8. This sequence of autoimmunity to different islet cell proteins indicates that destruction of insulin producing cells is progressing in a particular sequence. These autoantibodies can be used to estimate an individual's risk of developing type I diabetes [64]. Gardener et al. reported anti-islet cell antibodies in 83% of PCOS patients [63].

In PCOS low level of progesterone overstimulates immune system that leads to production of autoantibodies and therefore it can be labeled as an autoimmune disorder [61]. Summary of the autoantibodies that have been detected so far in PCOS is given in Table 3.

Females with PCOS are at increased risk for endometrial cancer, whereas their risks for breast and ovarian cancer are similar to those of women in the general population [65]. Nulliparity, obesity, and prolonged unopposed increase in estrogen are some of the health consequences of PCOS that are associated with cancer [66–69].  A study carried out by Barry found a significant threefold increase in risk for endometrial cancer among women with PCOS but no significant excess risks for either breast or ovarian cancer [70].

Females of PCOS present with complaints of infertility, which is often anovulatory in nature. These patients are often treated by conventional ovulation-induction medications, such as clomiphene citrate (CC) or gonadotropins, as first-line therapy. These agents increase risks for multiple pregnancies and in case of gonadotropins, there is an increased risk for ovarian hyperstimulation syndrome. Both of these drug modalities increase the risk for ovarian cyst formation, pain, and/or ovarian torsion. Metformin has been used extensively to treat infertility and pregnancy issues [71].

## 5. Insulin Resistance, Obesity, and Androgens as Potential Source of Autoimmunity in PCOS

PCOS is essentially a hormonal disorder and is buttressed by insulin resistance and hyperandrogenism [72, 73]. Recently Mezaal et al. have reported involvement of insulin directly or indirectly in the production of sex hormones. They reported many insertions, deletion, and substitutions of INS gene in PCOS patients by gene sequencing; they suggested that insulin resistance can affect sexual function adversely and may even cause PCOS [74]. The pathophysiologic linkage between PCOS and type 1DM has been declared as autoimmune phenomenon but it is still not established. Researchers have suggested that exogenous insulin given as the treatment of type 1DM may contribute to the development of PCOS in these patients [75]. Nonphysiologically administered exogenous insulin can potentially stimulate the production of androgens by ovaries [76]. Codner et al. postulated that aggressive insulin therapy may be accounted for the development of PCOS in females of type 1DM. They reported high frequency of hyperandrogenism and PCOS in patients of type 1DM, which appears to be associated with aggressive exogenous insulin therapy [77].

Obesity which is common in PCOS patients further increases the risk of insulin resistance which in turn causes hyperandrogenism and risk of developing PCOS [78, 79]. However, the relationship between hyperandrogenism and metabolic features of PCOS is still very controversial. So far differing findings have been reported for the pathogenic role of different types of androgens in patients of PCOS [80]. Considering literature, for the better understanding of pathophysiologic mechanisms of PCOS, emphasis should be given to the role of autoimmunity in PCOS and its relationship with obesity, insulin resistance, and hyperandrogenism. This will help to further enhance the knowledge of clinicians about suitable diagnostic, preventive, and curative interventions for women with PCOS.

## 6. Avenues for the Future Research and Conclusion

There is an association between PCOS and autoimmune diseases such as ANA and anti-TPO that have been documented in systemic lupus erythematosus and Hashimoto thyroiditis, respectively, and it is suspected that there are autoantibodies that might affect the long term clinical management of these patients. Therefore fluctuating levels of autoantibodies in different PCOS patients give us the way to open new chapter for future research on molecular level. This may lead to discovery of better treatment options for PCOS in near future.

Since thyroid autoimmune markers are present in patients of PCOS, therefore, PCOS patients should be investigated for autoimmune markers of thyroid. Further, disturbances of estrogen, progesterone, and thyroid profile are causative factors of gynaecological problems that eventually may lead to fetal loss and occurrence of endometrial, ovarian, and breast cancers.

Further studies are needed at genetic and molecular level to establish the contribution of autoimmunity in PCOS. It is hoped that, with the advancements of diagnostic facilities, researchers would be able to establish early markers for the diagnosis and monitoring of PCOS and its associated disorders and maybe for its better prognosis.

## Figures and Tables

**Table 1:** Frequency of different clinical features of PCOS.

Clinical signs and symptoms of PCOS	Frequencies
Oligomenorrhea	80–90% [28, 29]

Amenorrhea	30–40% [29]
	10–75% [30]

Hypergonadism	70% [31]

Polycystic ovaries	90% [32]

Hirsutism	50% [33]
	70% [31]

Acne	15%–30% [34]

Alopecia	<10% [35, 36]

Infertility	40% [37]

Overweight/obese^*∗*^	30%–70% [38]

^*∗*^The majority of PCOS patients were overweight (BMI = 25 and <30 kg/m^2^) to obese (BMI = 30 kg/m^2^), although one-third were of normal weight or even underweight.

**Table 2:** Clinical phenotypes represented by consensus guidelines for PCOS.

Criteria for PCOS	Consensus diagnostic points
(1) Androgen Excess Society (AES) 2006 reaffirmed 1990 NIH criteria (2) Amsterdam ESHRE/ASRM consensus (2010) reaffirmed Rotterdam 2003 criteria	(1) Hyperandrogenism (2) Oligoanovulation and polycystic ovaries (3) Exclusion of other androgen excess disorders [18]

**Table 3:** Reported autoantibodies in PCOS.

Autoantibodies	Documented findings of different studies
ANA	8.6% [43] *p* < 0.001 [46]

Anti-RO (SSA)	5.7% [43]

Anti-dsDNA	1.97% (*p* = 0.553) [52] *p* = <0.001 [46]

Anti-thyroglobulin	7.81% (*p* = 0.29) [52] *p* = 0.275 [53] *p* = 0.039 [54]

Anti-TPO	18.75 (*p* = 0.045) [52] 26.7% (*p* = 0.002) [54] *p* = 0.040 [53]

Anti-carbonic anhydrase-1	26% [57]

Anti-spermatic antibody	22.61% [58]

Anti-ovarian antibody	44% [59] IgG 27% (*p* < 0.0001) IgA 3% (*p* < 0.003) IgM 27% (*p* < 0.0003)

Islet cell antibody	53% [60]

GAD	44% [60]

Antibody to protein tyrosine phosphatase	16% [60]

Insulin autoantibodies	21% [60]

Anti-histone antibody	11.4% (*p* = 0.035) [61]

Anti-SM antibody	Positive (*p* = 0.44) [62]
